# Reply

**DOI:** 10.1002/hep4.2083

**Published:** 2022-08-26

**Authors:** Ann‐Sofi Duberg, Charlotte Lybeck, Anna Fält, Scott Montgomery, Soo Aleman

**Affiliations:** ^1^ Department of Infectious Diseases, School of Medical Sciences, Faculty of Medicine and Health Örebro University Orebro Sweden; ^2^ Clinical Epidemiology and Biostatistics, School of Medical Sciences Örebro University Orebro Sweden; ^3^ Clinical Epidemiology Division Karolinska Institutet Stockholm Sweden; ^4^ Department of Epidemiology and Public Health University College London London United Kingdom; ^5^ Department of Infectious Diseases Karolinska University Hospital Stockholm Sweden; ^6^ Department of Medicine Huddinge Karolinska Institutet Stockholm Sweden

We thank Dr. Singh and Dr. Mondia for their interest in our study on the risk of hepatocellular carcinoma (HCC) by age and country of origin in people with chronic hepatitis B virus (HBV) infection living in Sweden.

The highest incidences of HCC have been reported from HBV‐endemic regions, predominantly Asia and sub‐Saharan Africa, and a higher risk of very early onset HCC has been suggested in people from sub‐Saharan Africa.^[^ ^1^, ^2^, ^3^ ^]^ Clinical practice guidelines recommend HCC surveillance at younger ages for people infected with HBV without cirrhosis originating from Asia and Africa.^[^ ^4^, ^5^ ^]^ However, there are few studies of HCC incidence in people infected with HBV from different countries, particularly African‐born, who have immigrated to Europe or the United States.

Therefore, we wanted to study and compare the incidence of HCC by age and country of origin in people with chronic HBV infection in Sweden. About 90% are immigrants from HBV‐endemic areas, approximately as many people from East/Southeast Asia, sub‐Saharan Africa, Greater Middle East, and Europe (including Sweden). Many became Swedish residents with a personal identification number, making it possible for follow‐up in the universally available health care system using national registers with prospectively collected data: This makes Sweden a suitable country to study HBV‐related outcomes by region of origin.

One strength is that every resident with a diagnosis of HBV infection is included, and the national registers add information on date of immigration, country of birth, HCC, morbidity, and mortality. Possible limitations are mentioned in the article; for example, we lack results from blood tests including detailed virology, stage of liver fibrosis other than cirrhosis diagnosis, weight, alcohol consumption, and family history of HCC. This information would probably not be complete for a whole national cohort even with information from medical records. In addition, obesity and NAFLD are diagnoses that are often missing from records or underdiagnosed.

To conclude, we agree with Dr. Singh and Dr. Mondia that several other factors are associated with an increased risk of HCC, but it is not possible to include all possible risk factors in a register study of a national cohort with chronic HBV infection. All studies have their strengths and limitations, and different types of studies are needed to complement each other. Despite this, the findings from our study help in identifying sections of the population that may benefit from additional HCC screening.

## CONFLICT OF INTEREST

SA is on the speakers’ bureau for, and received grants from, Gilead and AbbVie, and is on the speakers’ bureau for MSD. ASD is on the speakers’ bureau for Gilead, MSD, and AbbVie.
